# Unravelling the multilayer growth of the fullerene C_60_ in real time

**DOI:** 10.1038/ncomms6388

**Published:** 2014-11-05

**Authors:** S. Bommel, N. Kleppmann, C. Weber, H. Spranger, P. Schäfer, J. Novak, S.V. Roth, F. Schreiber, S.H.L. Klapp, S. Kowarik

**Affiliations:** 1 Institut für Physik, Humboldt-Universität zu Berlin, Newtonstrasse 15, 12489 Berlin, Germany; 2 Deutsches Elektronen-Synchrotron (DESY), Notkestrasse 85, 22607 Hamburg, Germany; 3 Institut für Theoretische Physik, Technische Universität Berlin, Hardenbergstrasse 36, 10623 Berlin, Germany; 4 Institut für Angewandte Physik, Universität Tübingen, Auf der Morgenstelle 10, 72076 Tübingen, Germany

## Abstract

Molecular semiconductors are increasingly used in devices, but understanding of elementary nanoscopic processes in molecular film growth is in its infancy. Here we use real-time *in situ* specular and diffuse X-ray scattering in combination with kinetic Monte Carlo simulations to study C_60_ nucleation and multilayer growth. We determine a self-consistent set of energy parameters describing both intra- and interlayer diffusion processes in C_60_ growth. This approach yields an effective Ehrlich–Schwoebel barrier of *E*_ES_=110 meV, diffusion barrier of *E*_D_=540 meV and binding energy of *E*_B_=130 meV. Analysing the particle-resolved dynamics, we find that the lateral diffusion is similar to colloids, but characterized by an atom-like Schwoebel barrier. Our results contribute to a fundamental understanding of molecular growth processes in a system, which forms an important intermediate case between atoms and colloids.

## Abstract (web summary)

Despite the technological relevance of molecular thin films, there is limited understanding of their growth on a molecular level. Here, the authors characterize the relevant processes in real time and determine energy parameters using a combination of X-ray techniques and kinetic Monte Carlo simulations.

## Graphical abstract

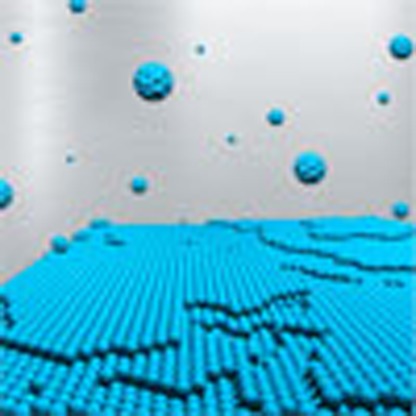

Understanding the growth of molecular materials such as the prototypical molecular semiconductor fullerene C_60_ (refs 1, 2) on surfaces is an indispensable prerequisite for the rational design of complex nanomaterials from molecular building blocks, as well as for optimizing the performance in thin-film-based applications such as solar cells^3^,^4^,^5^ and organic light-emitting diodes^6^,^7^. So far, molecular self-assembly and growth^8^ has often been described by scaling laws to describe surface roughening and evolving island densities^9^,^10^. On a molecular level, a range of studies have elucidated the kinetics of diffusion and nucleation (see, for example, refs 11, 12, 13, 14, 15, 16) and the Ehrlich–Schwoebel barrier for interlayer transport across a molecular step edge^11^,^17^,^18^ (see Fig. 1). In the last decades, the energy barriers for atomic growth have been refined to take into account the local neighbourhood during multilayer growth, for example, by including concerted gliding of islands or by distinguishing between different step-edge orientations^19^,^20^,^21^,^22^. Yet to date, there is no organic compound for which even the ‘minimal’ set of the three parameters diffusion barrier, lateral binding energy and Ehrlich–Schwoebel barrier have been simultaneously quantified to describe multilayer molecular growth. Therefore, predictive simulations of the rate- and temperature-dependent morphology in molecular multilayer growth have so far been impossible, contrary to the situation for elemental atomic systems^23^,^24^,^25^ and colloids^26^,^27^,^28^. Importantly, C_60_ exhibits properties in between those of atoms and colloids, which makes it a test case of fundamental relevance. On one hand, its van der Waals diameter of 1 nm^29^ is closer to atomic dimensions than to the μm length scale of colloidal systems. On the other hand, C_60_ resembles colloids with its short-range nature of the effective centre-of-mass interactions^30^, which decay as −1/*r*^9^ with *r* being the centre-of-mass separation stemming from the averaged van der Waals interactions (approximately −1/*r*^6^) between the individual carbon interaction sites^31^. These forces between atomic, molecular or colloidal building blocks are of prime importance for kinetic growth processes, similar to their role in equilibrium phase behaviour and self-assembly^32^,^33^. For example, C_60_ lacks a stable equilibrium liquid phase^30^, contrary to most elemental atomic systems. C_60_ is therefore not only relevant for device applications, but also an important, fundamentally unique material bridging atoms and colloids. From the experimental side, a particular challenge in studying C_60_ growth is that post-growth changes make the interruption of this non-equilibrium process to image different growth stages potentially misleading. It is therefore essential to use *in situ* real-time techniques.

In this article, we employ the combination of specular X-ray growth oscillations^34^ with real-time diffuse X-ray scattering^35^,^36^ to simultaneously follow the vertical and lateral morphology during growth. Further understanding on a nanoscale level is provided by kinetic Monte Carlo (KMC) simulations of coarse-grained C_60_ molecules without internal degrees of freedom. Then, the three relevant parameters determined by a fit of the data are the Ehrlich–Schwoebel barrier, the surface diffusion barrier and the lateral binding energy (see Fig. 1). With these parameters alone, we achieve quantitative agreement with the experimental data, enabling us to predict the rate-, temperature- and thickness dependency of the film morphology. Moreover, our analysis demonstrates that the short interaction range of C_60_ as compared with atoms affects the relative heights of diffusion barrier and binding energy and results in comparatively long diffusion times. However, unlike the colloidal systems, C_60_ has a true energetic Ehrlich–Schwoebel barrier, rather than the pseudobarrier that colloids display^26^.

## Results

### Experimental results for the layer-by-layer growth of C_60_ on mica

For a comprehensive understanding of the processes during growth, the surface morphology has to be measured on the molecular length scale with an experimental time resolution that is fast compared with the minute timescale of the deposition of a monolayer. Interrupting growth to take a series of real-space microscopy images can be problematic, as the kinetics can be altered. For our system of C_60_ on top of a closed first C_60_ layer on mica, this route is indeed impossible because of quick dewetting effects characterized by a time constant of ~10 min. Also, *in situ* low-energy electron microscopy unfortunately—while very successfully used in a range of studies^37^,^38^—cannot be applied due to charging effects on mica. Therefore, we use X-ray scattering that can be performed non-invasively during growth and yields time-resolved information about the layer formation. This is extracted through specular reflectivity measurements at the so-called anti-Bragg position of C_60_ (see Fig. 2a) corresponding to half the Bragg value 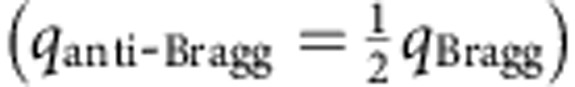 of the C_60_(111) reflection. Lateral information is available through simultaneous measurement of the diffuse scattering (grazing incidence small-angle X-ray scattering (GISAXS)), giving information about the island distance (Fig. 2a).

The time-dependent specular X-ray reflectivity as a function of molecular exposure, which is time × deposition rate, is shown in Fig. 2b for growth at *T*=60 °C substrate temperature and a deposition rate of *f*=0.1 ML min^−1^. The anti-Bragg intensity oscillates with a period of two monolayers (ML) as the X-rays are reflected from consecutive C_60_ layers and alternately interfere destructively and constructively with an intensity modulation of up to 90%. Here, the diffusely scattered intensity can be neglected in an analysis of the specular reflectivity, as it represents <1% of the total intensity. The oscillations are indicative of a layer-by-layer growth and from the change in oscillation period, a variation of the sticking coefficient is deduced (see Methods). Only after the first three layers, one observes a damping of the oscillations, reflecting the onset of slight roughening. An additional discussion on the anti-Bragg intensity during the growth of the first monolayer of C_60_ on mica is given in Supplementary Note 1 and illustrated in Supplementary Fig. 1.

While the diffuse scattering is weak, it nevertheless contains important lateral information. Figure 2c shows a map of the diffusely scattered intensity as a function of *q*_‖_ and molecular exposure (see Supplementary Fig. 2 for a graph of the diffusely scattered intensity at a molecular exposure of 0.3 nm). In contrast to the anti-Bragg oscillations, the diffusely scattered intensity oscillates with a period of one monolayer. As the first molecules are deposited in a monolayer, the surface roughness and therefore the diffusely scattered intensity rises due to nucleation of islands. Eventually, as the islands coalesce, the roughness and diffuse intensity decrease again, before reaching a minimum for a smooth complete layer. For each C_60_ layer, the diffusely scattered intensity has two maxima along *q*_‖_, because the characteristic average island distance *D* causes an increase in the diffuse scattered intensity at Δ*q*_‖_≈±2*π*/*D* (refs 39, 40).

From a crystallographic perspective, we find the established^41^ epitaxial order of C_60_ on top of mica(001) as confirmed by grazing incidence X-ray diffraction experiments shown in Supplementary Fig. 3 and explained in Supplementary Note 2.

### KMC simulations of the growth process

To understand the morphological evolution on a molecular level, we employ KMC simulations, which are capable of describing the entire growth process of (coarse-grained) C_60_ molecules into a face-centred cubic (fcc) lattice. KMC models the growth as a stochastic process, in which the molecules adsorb with a constant net adsorption rate *f*=*f*_adsorb_−*f*_desorb_. The molecules are treated on a coarse-grained level, that is, we do not take into account any internal (rotational or vibrational) degrees of freedom. This coarse-graining approach is supported by the fact that for the temperatures studied here, C_60_ rotates freely both in bulk crystals^42^ and in one-dimensional confinement^43^. Once adsorbed, a particle at site *i* then can diffuse to a neighbouring fcc site *j* via an activated process with Arrhenius-type rate *r*_*i,j*_. We follow the Clarke–Vvedensky bond-counting approach^44^,^45^, where the rate is defined as




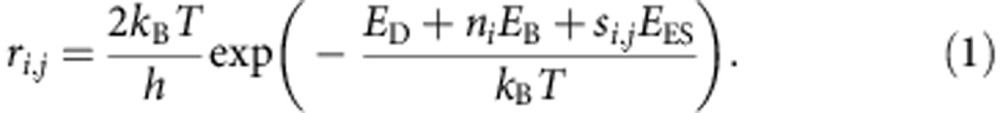




The pre-factor *v*_0_=2*k*_B_*T*/*h* is chosen in accordance with previous KMC studies for atomic systems^46^,^47^,^48^, consistent with our coarse-grained description of C_60_ as a sphere. The total energy barrier for molecular hopping consists of a barrier for free diffusion, *E*_D_, and contributions determined through the local neighbourhood of the particle. The neighbour binding energy *E*_B_ contributes with a number of lateral neighbours *n*_*i*_. The sum of *E*_D_ and *n*_*i*_*E*_B_ then determines the lateral diffusion (*s*_*i,j*_=0) and thus, the growth of islands. Other pre-factors to the neighbour binding energy have been suggested in literature^19^,^24^, which increase the diffusion rate of particles along island edges. As a consequence, the islands become more compact. In our C_60_ system, however, the islands are quite compact from the very onset of the growth (see Fig. 3). Therefore, the details of the pre-factor of *E*_B_ do not significantly influence the results at the parameters considered. If a particle at site *i* crosses an up- or downward step to reach site *j*, an additional Ehrlich–Schwoebel contribution *E*_ES_ is added to the total energy barrier (*s*_*i,j*_=1). As a result, a particle diffusing onto an island from an edge site with two neighbours has to overcome the activation energy Δ*E*=*E*_D_+2*E*_B_+*E*_ES_, while a particle on the island has to overcome only Δ*E*=*E*_D_+*E*_ES_ to diffuse downwards over the island edge. The step-edge energy barrier used in our simulations is, by construction, an average energy barrier. For this, we recall that our energy barriers are exclusively gained by comparison with experiment, and that the experimental (X-ray scattering) data are intrinsically averaged in lateral direction. Therefore, we did not take into account the orientation of the step edge in this study. The KMC input parameters *T* (substrate temperature) and *f* (adsorption rate) are taken directly from experiment. The KMC simulations have been performed from the second layer onwards as we concentrate on the C_60_–C_60_ interactions and do not model C_60_–mica interactions. This strategy is justified, as we know from the experiment that the first C_60_ layer is completely filled and that there is no lattice strain; thus, we can assume a smooth C_60_(111) surface as initial surface in simulations. Furthermore, we assume defect-free growth without cavities or overhangs. We also note that we do not take collective diffusion mechanisms into account. Different concepts for collective diffusion have been suggested in the literature, one example being dimer shearing^49^. More recently, approaches have been suggested for shearing, reptation and concerted gliding of islands^50^. These phenomena are certainly worth studying in more detail, however, it would not have been possible to simulate the time and length scale required in our study if these effects were included.

### Energy barriers for surface processes in C_60_ growth

For the comparison of experiment and simulations, we use the time-dependent layer coverages from KMC simulations to calculate anti-Bragg oscillations using kinematic scattering theory^51^ (see Methods). The energy barriers *E*_D_, *E*_B_ and *E*_ES_ (see equation (1)) are then adjusted until both the simulated anti-Bragg oscillations and island densities fit the experiment. Figure 3a,b shows experimental (black dots) and KMC simulation data (red solid line) for the island density and the anti-Bragg intensity for the temperature *T*=60 °C. The experimental island density 
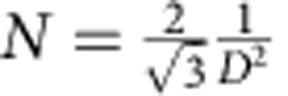
 is directly extracted from the data in Fig. 2c, using the average island distance *D*≈2*π*/Δ*q*_‖_, assuming a hexagonal island arrangement (see also Supplementary Fig. 4 and Supplementary Note 3 for a comparison with real-space atomic force microscope data). Both experiment and simulation predict that the island density changes markedly during the deposition of each monolayer. Initially, in the nucleation regime, the island density increases. Then, lateral island growth sets in, where the island density stays constant. Finally, the island density drops again as islands merge in the coalescence regime. The inset in Fig. 3a shows the corresponding KMC simulation snapshots for the three growth regimes. In all cases, we observe compact island shapes in the simulations as well as in the experiments. A more detailed comparison of the morphology is given in Supplementary Note 4 and shown in Supplementary Fig. 5. The sequence of growth regimes is observed for the first five layers at each temperature and deposition rate employed.

As it is clearly seen from Fig. 3a,b, there is excellent agreement between the experimental and simulated data regarding the island density and anti-Bragg growth oscillations. The minima and the maxima in the island density, as well as the trend of decreasing density for the different layers (increase in island size), are clearly reproduced. The apparent increase in the island density in the fifth layer, which starts to differ slightly from the true island density, indicates the limits of our data analysis. The analysis takes into account only the islands in a single, currently growing layer, however, due to the roughening of the film, both islands in the simultaneously growing 4th and the 5th layer contribute to the diffuse scattering at that stage. The vertical layer filling and roughening are also highly consistent, as can be seen from the good agreement between experimental and simulated evolution of anti-Bragg intensity in Fig. 3b. As an independent confirmation of the KMC results, we have employed a mean-field analytical model for thin-film growth (see refs 52, 53, 54), the results of which agree with the layer coverages of the KMC simulations, as can be seen in Fig. 3c.

**Figure 1:**
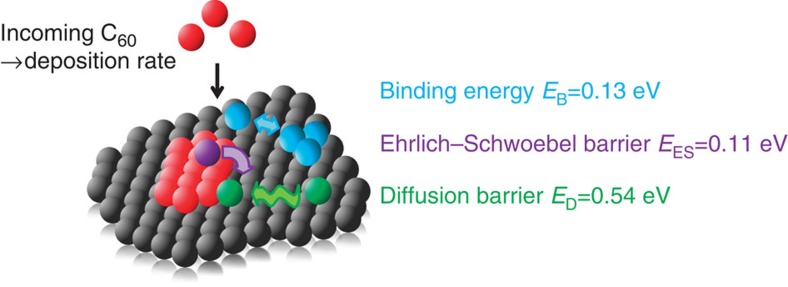
Surface processes in C_60_ growth. The diffusion barrier *E*_D_, binding energy *E*_B_ and Ehrlich–Schwoebel barrier *E*_ES_ determine island nucleation and interlayer transport in multilayer growth. Included are numerical values determined by fitting the experiment using KMC simulations.

**Figure 2:**
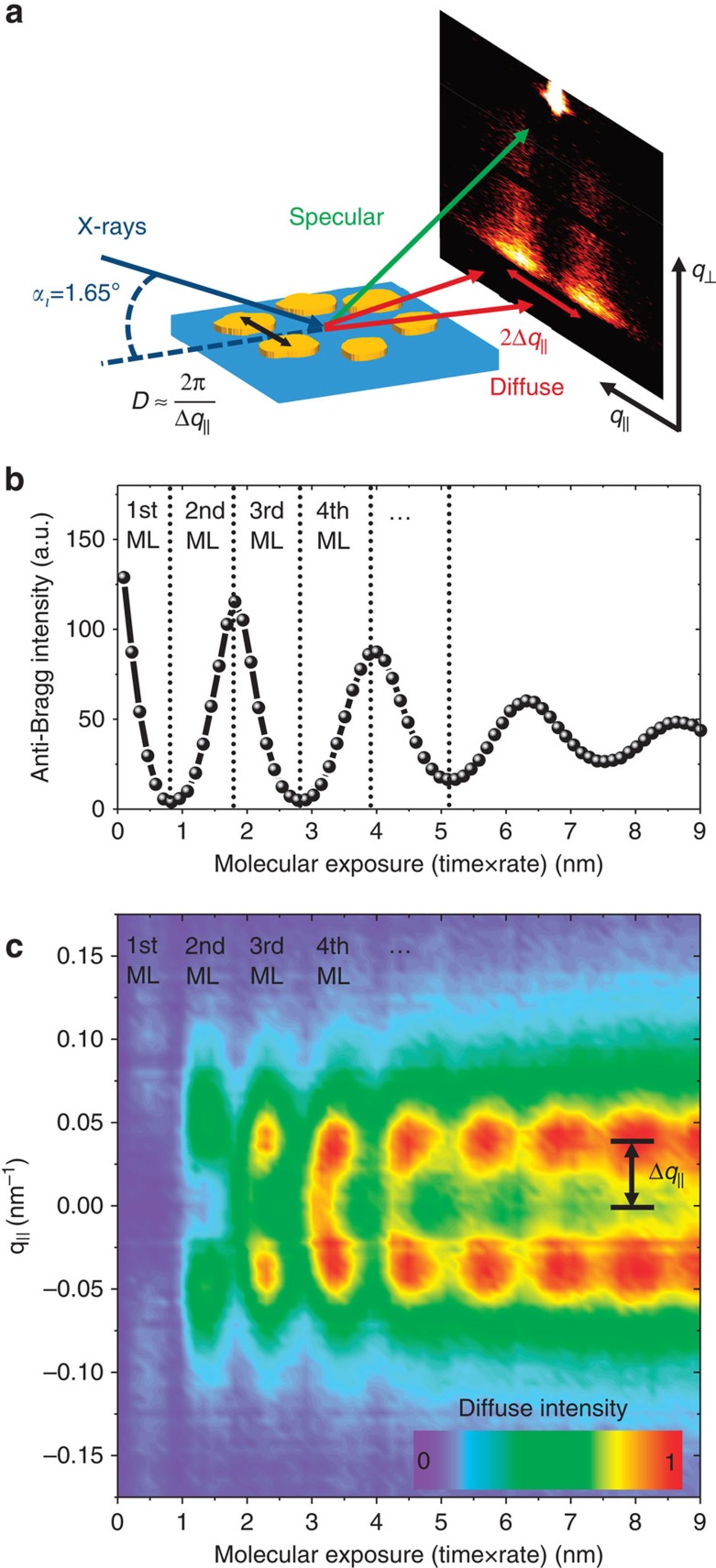
Specular and diffuse X-ray scattering during C_60_ growth. (**a**) Scattering geometry: both the specular reflected X-ray beam and the diffuse scattering are detected. The two-dimensional scattering pattern contains both lateral (transfer momentum *q*_‖_) and vertical (*q*_⊥_) information on the surface morphology. (**b**) The specular X-ray reflectivity at the anti-Bragg point *q*_⊥_=0.38 Å^−1^ oscillates with increasing molecular exposure (time × growth rate) during growth of C_60_ on mica indicating layer-by-layer growth (*T*=60 °C). (**c**) The diffusely scattered intensity oscillates with the nucleation and coalescence of every layer and exhibits a characteristic peak-splitting Δ*q*_‖_. The latter corresponds to the inverse average island distance, which changes with film thickness.

**Figure 3:**
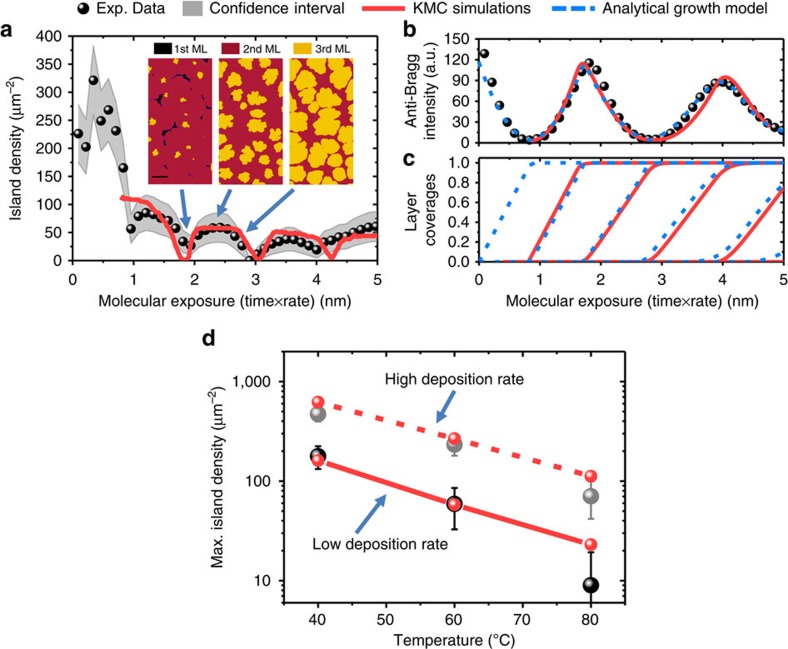
Experimental and simulated measures of surface morphology. (**a**) Island density (inset: 2D island growth regimes as simulated by KMC; scale bar, 100 nm), (**b**) anti-Bragg growth oscillations and (**c**) layer coverages are shown as a function of the molecular exposure for a C_60_ film grown at *T*=60** **°C and *f*=0.1** **ML min^−1^. Parts **b**,**c** include data from an analytical growth model. (**d**) Maximal island density for the third layer for both a low deposition rate of 0.1 ML min^−1^ and a high deposition rate of 1 ML min^−1^ as a function of temperature. The KMC simulations have been performed from the second layer onwards. The confidence interval in **a** and the error bars in **d** are calculated from the systematic experimental uncertainties. For the complete morphology evolution during growth for *T*=60 °C, *f*=0.1** **ML min^−1^ as well as 40 °C, 0.1 ML min^−1^ and 60 °C, 1 ML min^−1^ simulated by KMC, see Supplementary Movies 1–3.

Even beyond the specific experimental parameters chosen in Fig. 3a–c, KMC simulations show a good agreement with the experimental findings for all studied rates (0.1 and 1 ML min^−1^) and the full experimental temperature range of 40–80 °C (see Supplementary Note 5 and Supplementary Fig. 6 for a comparison of 40 °C and 0.1 ML min^−1^). This is seen in Fig. 3d, where we compare the experimental and simulated values for the maximum island density in the third monolayer. In accordance with growth theories predicting a scaling of island density with deposition rate/diffusivity^10^,^23^, we find that the island density decreases for higher substrate temperature and lower deposition rate by an order of magnitude. Furthermore, KMC simulations correctly predict the change in island density by an order of magnitude when changing deposition rate and temperature. Notably, this comprehensive agreement of temperature-, rate- and time-dependent data was achieved with a physical model of surface processes that contains only three parameters for the nanoscopic energy barriers for diffusion, nucleation and step-edge crossing. The resulting values are *E*_D_=(540±40) meV for the diffusion energy, *E*_B_=(130±20) meV for the lateral binding energy and *E*_ES_=(110±20) meV for the step-edge/Ehrlich–Schwoebel barrier (see also Fig. 1). For a more detailed discussion of the mutual correlations between energy parameters, see Supplementary Note 6.

## Discussion

It is instructive to compare the self-consistent parameter set obtained in this study to energy values reported earlier. The height of the C_60_ Ehrlich–Schwoebel barrier (110 meV) is comparable to atomic systems, such as Pt/Pt(111) (80 meV)^24^ and is close to the value of 100 meV for C_60_ from recent density functional theory calculations by Goose *et al.*^55^ Our value for the binding energy, *E*_B_=130 meV, is smaller than that related to the minimum of the pair interaction potential of two C_60_ molecules, in particular the Girifalco potential, *E*_C60–C60_=270 meV, which has been derived theoretically^56^,^57^ and has recently been measured in atomic force microscope experiments^58^. There are several factors contributing to this difference: first, we are considering molecules close to a substrate, which has not been taken into account in refs 56, 57 but has already been shown to weaken the interaction^58^. Second, we are considering dense and thus strongly correlated systems, not two molecules in vacuum as assumed in refs 56, 57. Third, and maybe most importantly, our value for the binding energy has been obtained such that experimental data are fitted over a range of temperatures. It is well known that effective potentials (and thus binding energies) can strongly depend on the temperature^59^; thus our value has to be considered as a temperature average. Finally, we stress that our value for *E*_B_ is very close to an estimate gained from the cohesion energy per neighbour of C_60_ in its bulk fcc crystal, *E*_C_=133 meV (1.6 eV is the total cohesion energy^60^,^61^ divided by the 12 bulk lattice neighbours). Regarding our value for the diffusion barrier (*E*_D_=540 meV), we note that this is significantly larger than the corresponding value derived from a potential landscape analysis, *E*_pot_=168 meV (ref. 62). This is likely due to the fact that in our KMC simulations, we do not consider all energy minima as lattice sites. Thus, the travelled distances across several minima are larger, leading effectively to a larger barrier. In addition, we cannot exclude stacking faults and domains in the epitaxial C_60_ adlayers, which could contribute to a larger effective diffusion barrier in our calculation as transport across domain boundaries is hindered. A more detailed comparison of our value for the diffusion barrier with values derived from pair potential calculations and molecular dynamics simulations is given in Supplementary Note 7. Without this strategy, the simulation of the full multilayer growth would have been impossible. Furthermore, the same strategy is used in simulations of metallic growth^24^,^63^,^64^ enabling a comparison with these studies.

**Figure 4:**
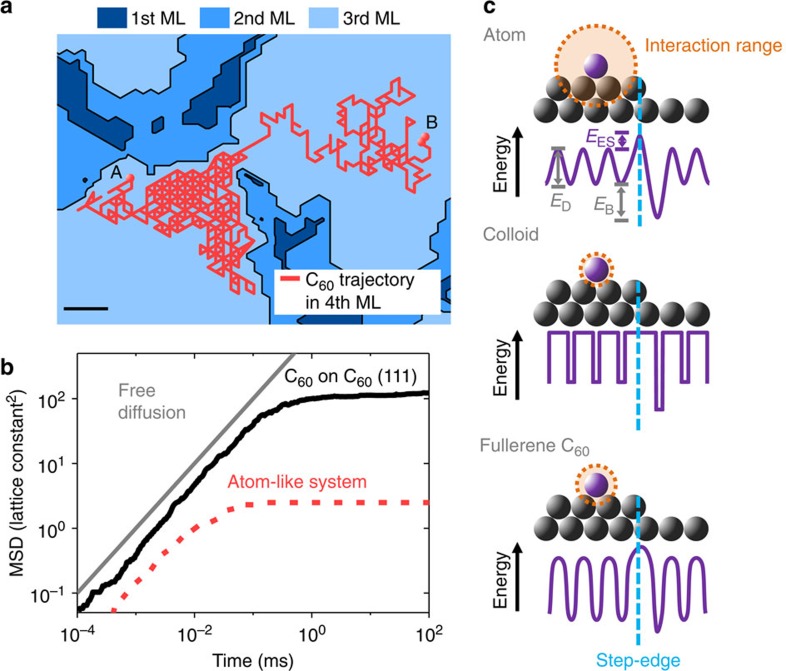
Particle-resolved dynamics during C_60_ growth. (**a**) Trajectory of a single molecule in the 4th ML (*T*=40 °C and *f*=1 ML min^−1^; scale bar, 5 nm). The influence of the Ehrlich–Schwoebel barrier can be clearly seen as a caging of the single C_60_ molecule on the island. The letters A and B denote the adsorption of one molecule on the surface (A) and the formation of a dimer (B). (**b**) MSD=‹|**r**(*t*)−**r**(0)|^2^› of C_60_ on C_60_(111), for *T*=60 °C and *f*=0.1 ML min^−1^ as a function of time spent on the surface. Results are averaged over 500 realizations. The particles considered arrive in the 2nd ML after the growth of 1.5 monolayers. For comparison, we show data for a system with atom-like ratio *E*_D_/*E*_D_+*E*_B_=0.34. Note that the quasi-free diffusion of C_60_ extends substantially further than for atom-like systems, even if scaled by the lattice parameter, signifying the qualitatively different behaviour of C_60_. (**c**) Schematic illustration of energy landscape for atoms, colloids and the fullerene C_60_ near an island step edge: The interaction range of the different materials clearly affects the character of step-edge barrier as one can distinguish between real and a diffusion-mediated pseudobarrier^26^.

In addition to the quantities discussed so far, KMC simulations allow us to extract single-particle trajectories and, thus, to study the dynamics on a particle level, which is not yet possible with current experimental techniques. An example of a single C_60_ particle trajectory (red) on top of a third monolayer island (light blue) is shown in Fig. 4a. Clearly, the Ehrlich–Schwoebel barrier leads to a ‘caging’ of the C_60_ molecule close to the borders of the island, that is, the standard random walk behaviour is restricted by the step edge of the island.

Importantly, the particle-resolved dynamics reveal crucial differences in the diffusion behaviour of C_60_ and atomic systems. For C_60_ on C_60_(111), the diffusion barrier *E*_D_ is relatively large compared with the binding energy *E*_B_. Specifically, the ratio *R*=*E*_D_/(*E*_D_+*E*_B_) is *R*=0.83. This is significantly larger than in typical atomic systems, such as Pt on Pt(111) where *R*≈0.29–0.34, or Ag on Ag(111) with *R*≈0.29–0.39 (refs 23, 24). We suggest that this pronounced difference is related to the relatively short attractive interaction range of C_60_, as compared with the attraction range of atoms, if normalized to their respective size (for details see Supplementary Fig. 7 and Supplementary Notes 8 and 9). The comparatively large ratio *R* for C_60_ has a profound impact on the mobility of the particles. This is shown in Fig. 4b, where we plot the mean-squared displacement, MSD=‹|**r**(*t*)−**r**(0)|^2^› for particles arriving between islands after the growth of 1.5 monolayers for C_60_ and for a system with an atom-like ratio *R*=0.34.

The linear increase with time of the C_60_ MSD in the very beginning corresponds to free diffusion, depicted in grey, as the molecules perform a random walk on the underlying fcc(111) surface. After a time of about 0.1 ms, encountering an upward island edge as well as interactions with neighbours hinder the diffusion of the molecules, the MSD saturates. Similar sub-diffusive behaviour also occurs in the atom-like system, but at much shorter times. This is because atoms can form new bonds more quickly due to the longer range of atomic interactions and the stronger binding energy. As a result, a C_60_ molecule is able to explore an area that is nearly two orders of magnitude larger than in the atom-like system before it is immobilized.

The different diffusion behaviour of C_60_ prompts the question on the nature of the Ehrlich–Schwoebel barrier in comparison with atomic and colloidal growth. Indeed, regarding their narrow interaction range, C_60_ ‘nanocolloids’ are more similar to colloids than atoms. In colloids, the range of attractive interactions is so small that the reduced coordination associated with an edge is not ‘sensed’. This effectively leads to the vanishing of an energetic barrier at the edge. Instead, one observes a purely diffusive Ehrlich–Schwoebel barrier in colloids, arising from a lower diffusion probability along the geometrically longer path across the step edge^26^. In contrast, atoms crossing an island edge have to overcome an energetic Ehrlich–Schwoebel barrier, as bonds are missing at the step-edge. For C_60_, we can estimate an upper bound for a diffusive barrier based on the waiting time of a typical hopping process. Multiplying this time by a geometric factor (see ref. 26), which accounts for the longer path of a step-edge crossing, we obtain a diffusive pseudobarrier of *E*_ES,geo_=ln(*F*) *k*_B_*T*<50 meV (see Supplementary Note 10 and Supplementary Fig. 8 for details). This is markedly smaller than the value of 110 meV obtained from the KMC simulations. We thus conclude that the Ehrlich–Schwoebel barrier in C_60_ surface growth is, at least partially, of energetic character, consistent with the intermediate range of the C_60_ interactions (which lies between the range of colloidal and atomic interactions). This is schematically shown in the energy landscapes for atoms, colloids and C_60_ in Fig. 4c.

In conclusion, the present experimental and theoretical study yields, for the first time, a quantitative description of molecular thin-film growth for the important case of C_60_, as an intermediate between atoms and colloids. We have demonstrated that *in situ* specular X-ray reflectivity and diffuse GISAXS oscillations are powerful tools for non-invasive real-time studies of the morphological evolution during molecular growth. Relating the experimental data to results from KMC simulations, we have been able to determine a consistent set of energy parameters determining the growth kinetics on the molecular level. This way we can quantitatively predict C_60_ deposition at different temperatures and rates, including the evolution of island density and surface roughening with film thickness. Thus, our combined analysis provides a detailed understanding of C_60_ in terms of molecular-scale processes. Moreover, our study sheds new light on various dynamical aspects accompanying the growth. In particular, we show that the colloid-like, short-ranged character of C_60_ interactions leads to relatively long surface diffusion times before immobilization occurs at existing islands. Nevertheless, the step-edge crossing barrier of C_60_ differs from colloids in that it is not a pseudo-step-edge barrier arising from lower diffusion probability at a step edge, but a true energetic barrier as observed for atoms. Since C_60_ features aspects of both atomic and colloidal systems, our findings will help to gain insight into island nucleation and surface growth processes for van der Waals-bound molecules between the scales of atomic and colloidal systems. This quantitative, scale-bridging understanding enables predictive simulations and a rational choice of growth conditions, which, together with molecular design and synthesis, ultimately leads to optimized design of functional materials.

## Methods

### X-ray surface scattering and thin-film preparation

The X-ray surface scattering experiments during growth were carried out at the MiNaXS beamline P03 (ref. 65) of PETRA III (DESY, Hamburg) at an X-ray wavelength of 0.946 Å. The growth was performed in a portable ulta-high vacuum (UHV) chamber designed for molecular beam deposition, equipped with a Be window for X-ray access, C_60_ effusion cell and a quartz crystal microbalance, at a base pressure of 10^−8^ mbar. Fullerene C_60_ (Sigma Aldrich, >99.5% purity) was thermally deposited on cleaved mica (diameter: 10 mm, Plano GmbH) for two different deposition rates (0.1 and 1 ML min^−1^) and for three different substrate temperatures (40, 60 and 80 °C) to study rate-, temperature- as well as time- and thickness dependency of the island density and layer coverage. Films were grown repeatedly on the same substrate after heating the mica substrate to ~450 °C, resulting in a clean substrate, as confirmed by specular and diffuse X-ray scattering before every growth run. The high brilliance of the beamline and high dynamic range of the PILATUS 300 K (Dectris) area detector enable a simultaneous measurement of the strong specular X-ray reflectivity and weak diffuse X-ray scattering. An incident angle of *α*_*i*_=1.65°, the so-called anti-Bragg position of C_60_ corresponding to half the Bragg value 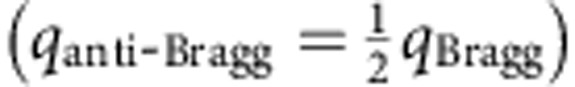 of the (111) reflection, was chosen. Here the reflectivity shows time-dependent oscillations during layer growth, which provide information on the vertical layer filling^16^,^53^. Lateral information is available through simultaneous measurement of the diffuse scattering (GISAXS), giving information about the island distance^39^, as a function of the lateral momentum of transfer *q*_‖_ at a resolution in *q*_‖_ of 0.001 Å^−1^. We avoided beam damage due to the high photon flux at PETRA III by laterally moving the substrate during the real-time growth experiments and confirmed that pristine and previously exposed spots gave the same scattering pattern in post-growth experiments.

### Anti-Bragg intensity and sticking coefficient

The time-dependent anti-Bragg intensity can be calculated in kinematic approximation using




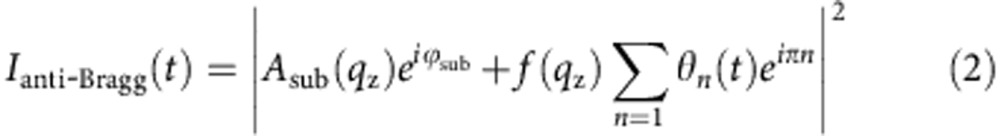




with the layer coverages *θ*_*n*_ for the *n*th layer. The substrate amplitude *A*_sub_, the substrate phase *ϕ*_sub_ and the molecular form factor *f*(*q*_z_) are determined by maximal, minimal and saturation intensity of the real-time experiment^51^. The anti-Bragg intensity for the KMC simulations was calculated using equation (2) and the simulated layer coverages 
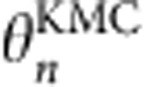
 shown in Fig. 4c. Furthermore, we have fitted the experimental data according to analytical growth models^51^,^53^ to extract the coverage evolution for each layer. In addition, we can extract the sticking coefficient from the anti-Bragg growth oscillations, which is found to decrease during the growth of the first four layers for all studied temperatures. Quantitatively, we find for a temperature of 60 °C that with respect to the growth of the first monolayer, the sticking coefficient decreases by 5% in the 2nd ML, 25% in the 3rd ML and 30% from the 4th layer onwards. This decrease is due to the different mica–C_60_ and C_60_–C_60_ interactions. It is further influenced by a different island density in each layer, which leads to a change in the free diffusion times and aggregation behaviour. In our KMC simulations, which otherwise assume complete condensation, we have accounted for the changing sticking coefficient by scaling the molecular exposure axis accordingly. The same sticking coefficients have also been included in our analytical mean-field modelling.

### Time step in KMC simulations

Assuming that exactly one process takes place in one simulated time step, we can define an average time-step length as




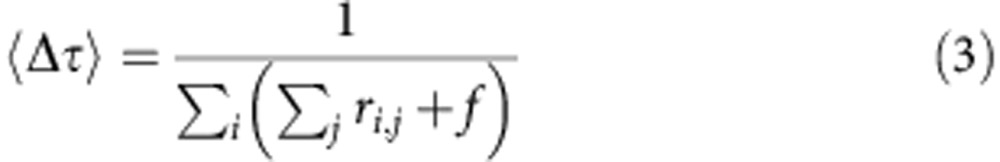




This time unit allows us to compare simulated and experimental timescales. The simulation is carried out on a triangular lattice. In this way, the growth process generates a fcc structure in accordance with the C_60_ bulk crystal (see the studies of Cox *et al.*^22^ for a similar simulation strategy for the growth of Ag on Ag(111), and of Heinrichs *et al.*^66^ for corresponding theoretical considerations). Starting point of the simulation is a completely filled, defect-free layer of C_60_ molecules (corresponding to the C_60_(111) surface). Within the subsequent growth process, we exclude the formation of overhangs. To achieve this, we assume that particles on overhang sites relax instantaneously (with a relaxation probability proportional to the corresponding diffusion rate) until they reach a stable site. Typical simulations involve a lattice with 1,000 × 1,000 unit cells, and they cover a time range up to 4,000 s, corresponding to (10^11^–10^12^) events.

## Author contributions

S.K., S.H.L.K. and F.S. proposed the research. S.B., C.W., P.S., H.S., J.N., S.V.R. and S.K. carried out the experiments, N.K. and S.H.L.K. performed the simulations and S.B. and N.K. analysed and fitted the data. All authors contributed to the writing of the manuscript.

## Additional information

**How to cite this article**: Bommel, S. *et al.* Unravelling the multilayer growth of the fullerene C_60_ in real-time. *Nat. Commun.* 5:5388 doi: 10.1038/ncomms6388 (2014).

## Supplementary Material

Supplementary Figures, Supplementary Notes and Supplementary References. Supplementary Figures 1-8, Supplementary Notes 1-10 and Supplementary References.

Supplementary Movie 1 Morphology evolution during C_60_ growth for T=60 °C and f=0.1 ML min^-1^ at 100 times the real speed.

Supplementary Movie 2 Morphology evolution during C_60_ growth for T=40 °C and f=0.1 ML min^-1^ at 100 times the real speed.

Supplementary Movie 3 Morphology evolution during C_60_ growth for T=60 °C f=1 ML min^-1^ at 10 times the real speed.

## References

[b1] Heath J., O’Brien S. & Curl R. 25 years of C_60_. Nat. Nanotech. 5, 691 (2010). 10.1038/nnano.2010.210 20924386

[b2] Dunk P. W. *et al.* Closed network growth of fullerenes. Nat. Commun. 3, 855 (2012). 22617295 10.1038/ncomms1853

[b3] Campoy-Quiles M. *et al.* Morphology evolution via self-organization and lateral and vertical diffusion in polymer:fullerene solar cell blends. Nat. Mater. 7, 158–164 (2008). 18204451 10.1038/nmat2102

[b4] Li Z. *et al.* Performance enhancement of fullerene-based solar cells by light processing. Nat. Commun. 4, 2227 (2013). 23892424 10.1038/ncomms3227

[b5] Schmidt-Hansberg B. *et al.* Moving through the phase diagram: morphology formation in solution cast polymer–fullerene blend films for organic solar cells. ACS Nano 5, 8579–8590 (2011). 22004659 10.1021/nn2036279

[b6] Brütting W. & Adachi C. Physics of Organic Semiconductors Wiley-VCH (2012).

[b7] Li N. *et al.* Efficient and bright organic light-emitting diodes on single-layer graphene electrodes. Nat. Commun. 4, 2294–2301 (2013). 23934428 10.1038/ncomms3294

[b8] Witte G. & Wöll C. Growth of aromatic molecules on solid substrates for applications in organic electronics. J. Mater. Res. 19, 1889–1916 (2004).

[b9] Dürr A. *et al.* Rapid roughening in thin film growth of an organic semiconductor (diindenoperylene). Phys. Rev. Lett. 90, 016104 (2003). 12570630 10.1103/PhysRevLett.90.016104

[b10] Ruiz R. *et al.* Dynamic scaling, island size distribution, and morphology in the aggregation regime of submonolayer pentacene films. Phys. Rev. Lett. 91, 136102 (2003). 14525320 10.1103/PhysRevLett.91.136102

[b11] Hlawacek G. *et al.* Characterization of step-edge barriers in organic thin-film growth. Science 321, 108–111 (2008). 18599783 10.1126/science.1159455

[b12] Liu H., Lin Z., Zhigilei L. V. & Reinke P. Fractal structures in fullerene layers: simulation of the growth process. J. Phys. Chem. C 112, 4687–4695 (2008).

[b13] Loske F., Lübbe J., Schütte J., Reichling M. & Kühnle A. Quantitative description of C_60_ diffusion on an insulating surface. Phys. Rev. B 82, 155428 (2010).

[b14] Körner M. *et al.* Second-layer induced island morphologies in thin-film growth of fullerenes. Phys. Rev. Lett. 107, 016101 (2011). 21797552 10.1103/PhysRevLett.107.016101

[b15] Einax M., Dieterich W. & Maass P. Colloquium: Cluster growth on surfaces: densities, size distributions, and morphologies. Rev. Mod. Phys. 85, 921–939 (2013).

[b16] Krause B., Schreiber F., Dosch H., Pimpinelli A. & Seeck O. H. Temperature dependence of the 2D-3D transition in the growth of PTCDA on Ag(111): a real-time X-ray and kinetic Monte Carlo study. Europhys. Lett. 65, 372–378 (2004).

[b17] Fendrich M. & Krug J. Ehrlich-Schwoebel effect for organic molecules: direct calculation of the step-edge barrier using empirical potentials. Phys. Rev. B 76, 121302 (2007).

[b18] Mayer A. *et al.* Growth dynamics of pentacene thin films: Real-time synchrotron X-ray scattering study. Phys. Rev. B 73, 205307 (2006).

[b19] Voter A. F. inRadiation Effects in Solids, NATO Science Series Vol. 235 (eds Sickafus K. E., Kotomin E. A., Uberuaga B. P. 1–23 (Springer (2007).

[b20] Teichert C., Ammer C. & Klaua M. Step formation on the ion-bombarded Ag(100) surface studied by LEED and Monte Carlo simulations. Phys. Status Solidi 146, 223–242 (1994).

[b21] Kürpick U. & Rahman T. Diffusion processes relevant to homoepitaxial growth on Ag (100). Phys. Rev. B 57, 2482–2492 (1998).

[b22] Cox E. *et al.* Temperature dependence of island growth shapes during submonolayer deposition of Ag on Ag(111). Phys. Rev. B 71, 115414 (2005).

[b23] Michely T. & Krug J. Islands, Mounds, and Atoms: Patterns and Processes in Crystal Growth Far From Equilibrium (Springer Series in Surface Sciences) Springer (2003).

[b24] Evans J. W., Thiel P. A. & Bartelt M. C. Morphological evolution during epitaxial thin film growth: formation of 2D islands and 3D mounds. Surf. Sci. Rep. 61, 1–128 (2006).

[b25] Ferguson J. D., Arikan G., Dale D. S., Woll A. R. & Brock J. D. Measurements of surface diffusivity and coarsening during pulsed laser deposition. Phys. Rev. Lett. 103, 256103 (2009). 20366266 10.1103/PhysRevLett.103.256103

[b26] Ganapathy R., Buckley M. R., Gerbode S. J. & Cohen I. Direct measurements of island growth and step-edge barriers in colloidal epitaxy. Science 327, 445–448 (2010). 20093469 10.1126/science.1179947

[b27] Bohlein T., Mikhael J. & Bechinger C. Observation of kinks and antikinks in colloidal monolayers driven across ordered surfaces. Nat. Mater. 11, 126–130 (2012). 22179397 10.1038/nmat3204

[b28] Frenkel D. & Wales D. J. Colloidal self-assembly: designed to yield. Nat. Mater. 10, 410–411 (2011). 21602872 10.1038/nmat3037 PMC3894446

[b29] Khlobystov A., Britz D., Ardavan A. & Briggs G. Observation of ordered phases of fullerenes in carbon nanotubes. Phys. Rev. Lett. 92, 245507 (2004). 15245099 10.1103/PhysRevLett.92.245507

[b30] Hagen M. H. J., Meijer E. J., Mooij G. C. A. M., Frenkel D. & Lekkerkerker H. N. W. Does C_60_ have a liquid phase? Nature 365, 425–426 (1993).

[b31] Tewari S. P., Dhingra G. & Silotia P. Collective dynamics of a nano-fluid: fullerene, C_60_. Int. J. Mod. Phys. B 24, 4281–4292 (2010).

[b32] Schmidle H., Jäger S., Hall C. K., Velev O. D. & Klapp S. H. L. Two-dimensional colloidal networks induced by a uni-axial external field. Soft Matter 9, 2518–2524 (2013).

[b33] Klapp S. H. L., Grandner S., Zeng Y. & von Klitzing R. Charged silica suspensions as model materials for liquids in confined geometries. Soft Matter 6, 2330–2336 (2010).

[b34] Kowarik S. *et al.* Real-time observation of structural and orientational transitions during growth of organic thin films. Phys. Rev. Lett. 96, 125504 (2006). 16605925 10.1103/PhysRevLett.96.125504

[b35] Müller-Buschbaum P. Grazing incidence small-angle X-ray scattering: an advanced scattering technique for the investigation of nanostructured polymer films. Anal. Bioanal. Chem. 376, 3–10 (2003). 12734612 10.1007/s00216-003-1869-2

[b36] Holý V. & Baumbach T. Nonspecular X-ray reflection from rough multilayers. Phys. Rev. B 49, 10668–10676 (1994). 10.1103/physrevb.49.10668 10009895

[b37] Meyer zu Heringdorf F. J., Reuter M. C. & Tromp R. M. Growth dynamics of pentacene thin films. Nature 412, 517–520 (2001). 11484047 10.1038/35087532

[b38] Khokhar F. S. *et al.* The influence of substrate temperature on growth of para-sexiphenyl thin films on Ir{111} supported graphene studied by LEEM. Surf. Sci. 606, 475–480 (2012). 22308005 10.1016/j.susc.2011.11.012 PMC3267044

[b39] Renaud G. *et al.* Real-time monitoring of growing nanoparticles. Science 300, 1416–1419 (2003). 12775836 10.1126/science.1082146

[b40] Schwartzkopf M. *et al.* From atoms to layers: *in situ* gold cluster growth kinetics during sputter deposition. Nanoscale 5, 5053–5062 (2013). 23640164 10.1039/c3nr34216f

[b41] Henke S., Thürer K. & Geier S. X-ray pole-figure study of the epitaxial growth of C_60_ thin films on mica (001). Appl. Phys. A 389, 383–389 (1995).

[b42] Heiney P. A. *et al.* Orientational ordering transition in solid C_60_. Phys. Rev. Lett. 66, 2911–2914 (1991). 10043651 10.1103/PhysRevLett.66.2911

[b43] Rols S. *et al.* How confinement affects the dynamics of C_60_ in carbon nanopeapods. Phys. Rev. Lett. 101, 065507 (2008). 18764476 10.1103/PhysRevLett.101.065507

[b44] Clarke S. & Vvedensky D. D. Growth kinetics and step density in reflection high-energy electron diffraction during molecular-beam epitaxy. J. Appl. Phys. 63, 2272–2283 (1988).

[b45] Oliveira T. J. & Aarão Reis F. D. A. Scaling in reversible submonolayer deposition. Phys. Rev. B 87, 235430 (2013).

[b46] Gyure M., Zinck J., Ratsch C. & Vvedensky D. Unstable growth on rough surfaces. Phys. Rev. Lett. 81, 4931–4934 (1998).

[b47] Jones A. *et al.* Faceting at the step flow threshold in epitaxial growth on patterned surfaces. Phys. Rev. B 79, 205419 (2009).

[b48] Marmorkos I. & Sarma S. Atomistic numerical study of molecular-beam-epitaxial growth kinetics. Phys. Rev. B 45, 262–272 (1992). 10.1103/physrevb.45.11262 10001050

[b49] Shi Z., Zhang Z., Swan A. & Wendelken J. Dimer shearing as a novel mechanism for cluster diffusion and dissociation on metal (100) surfaces. Phys. Rev. Lett. 76, 4927–4930 (1996). 10061415 10.1103/PhysRevLett.76.4927

[b50] Shah S. I., Nandipati G., Kara A. & Rahman T. S. Extended pattern recognition scheme for self-learning kinetic Monte Carlo simulations. J. Phys. Condens. Matter 24, 354004 (2012). 22898941 10.1088/0953-8984/24/35/354004

[b51] Kowarik S., Gerlach A., Skoda M. W. A., Sellner S. & Schreiber F. Real-time studies of thin film growth: measurement and analysis of X-ray growth oscillations beyond the anti-Bragg point. Eur. Phys. J. Spec. Top. 167, 11–18 (2009).

[b52] Trofimov V. I. & Mokerov V. G. Rate equations model for layer epitaxial growth kinetics. Thin Solid Films 428, 66–71 (2003).

[b53] Woll A. R., Desai T. V. & Engstrom J. R. Quantitative modeling of in situ X-ray reflectivity during organic molecule thin film growth. Phys. Rev. B 84, 075479 (2011).

[b54] Weber C. *et al.* Chain-length dependent growth dynamics of n-alkanes on silica investigated by energy-dispersive X-ray reflectivity in situ and in real-time. J. Chem. Phys. 136, 204709 (2012). 22667583 10.1063/1.4719530

[b55] Goose J. E., First E. L. & Clancy P. Nature of step-edge barriers for small organic molecules. Phys. Rev. B 81, 205310 (2010).

[b56] Girifalco L. Interaction potential for C_60_ molecules. J. Phys. Chem. 2, 5370–5371 (1991).

[b57] Pacheco J. & Prates Ramalho J. First-principles determination of the dispersion interaction between fullerenes and their intermolecular potential. Phys. Rev. Lett. 79, 3873–3876 (1997).

[b58] Chiutu C. *et al.* Precise orientation of a single C_60_ molecule on the tip of a scanning probe microscope. Phys. Rev. Lett. 108, 268302 (2012). 23005019 10.1103/PhysRevLett.108.268302

[b59] Babadi M., Everaers R. & Ejtehadi M. R. Coarse-grained interaction potentials for anisotropic molecules. J. Chem. Phys. 124, 174708–174718 (2006). 16689591 10.1063/1.2179075

[b60] Saito S. & Oshiyama A. Cohesive mechanism and energy bands of solid C_60_. Phys. Rev. Lett. 66, 2637–2640 (1991). 10043573 10.1103/PhysRevLett.66.2637

[b61] Troullier N. & Martins J. L. Structural and electronic properties of C_60_. Phys. Rev. B 46, 1754–1765 (1992). 10.1103/physrevb.46.1754 10003824

[b62] Gravil P. A. *et al.* Adsorption of C_60_ molecules. Phys. Rev. B 53, 1622–1629 (1996). 10.1103/physrevb.53.1622 9983626

[b63] Chen S., Liang J., Mo Y., Luo D. & Jiang S. Onset of shadowing-dominated growth of Ag films in glancing angle deposition: Kinetic Monte Carlo simulation. Appl. Surf. Sci. 264, 552–556 (2013).

[b64] Latz A., Brendel L. & Wolf D. E. A three-dimensional self-learning kinetic Monte Carlo model: application to Ag(111). J. Phys. Condens. Matter 24, 485005 (2012). 23099317 10.1088/0953-8984/24/48/485005

[b65] Buffet A. *et al.* P03, the microfocus and nanofocus X-ray scattering (MiNaXS) beamline of the PETRA III storage ring: the microfocus endstation. J. Synchrotron Radiat. 19, 647–653 (2012). 22713902 10.1107/S0909049512016895 PMC3380660

[b66] Heinrichs S., Rottler J. & Maass P. Nucleation on top of islands in epitaxial growth. Phys. Rev. B 62, 8338–8359 (2000).

