# Supplementary material for: Unravelling the multilayer growth of the fullerene C60 in real time
Source: Nat Commun. 2014 Nov 5;5:5388. doi: 10.1038/ncomms6388 (PMC4272254; doi:10.1038/ncomms6388)
Supplement: Supplementary Figures, Supplementary Notes and Supplementary References. — Supplementary Figures 1-8, Supplementary Notes 1-10 and Supplementary References. [file ncomms6388-s1.pdf]

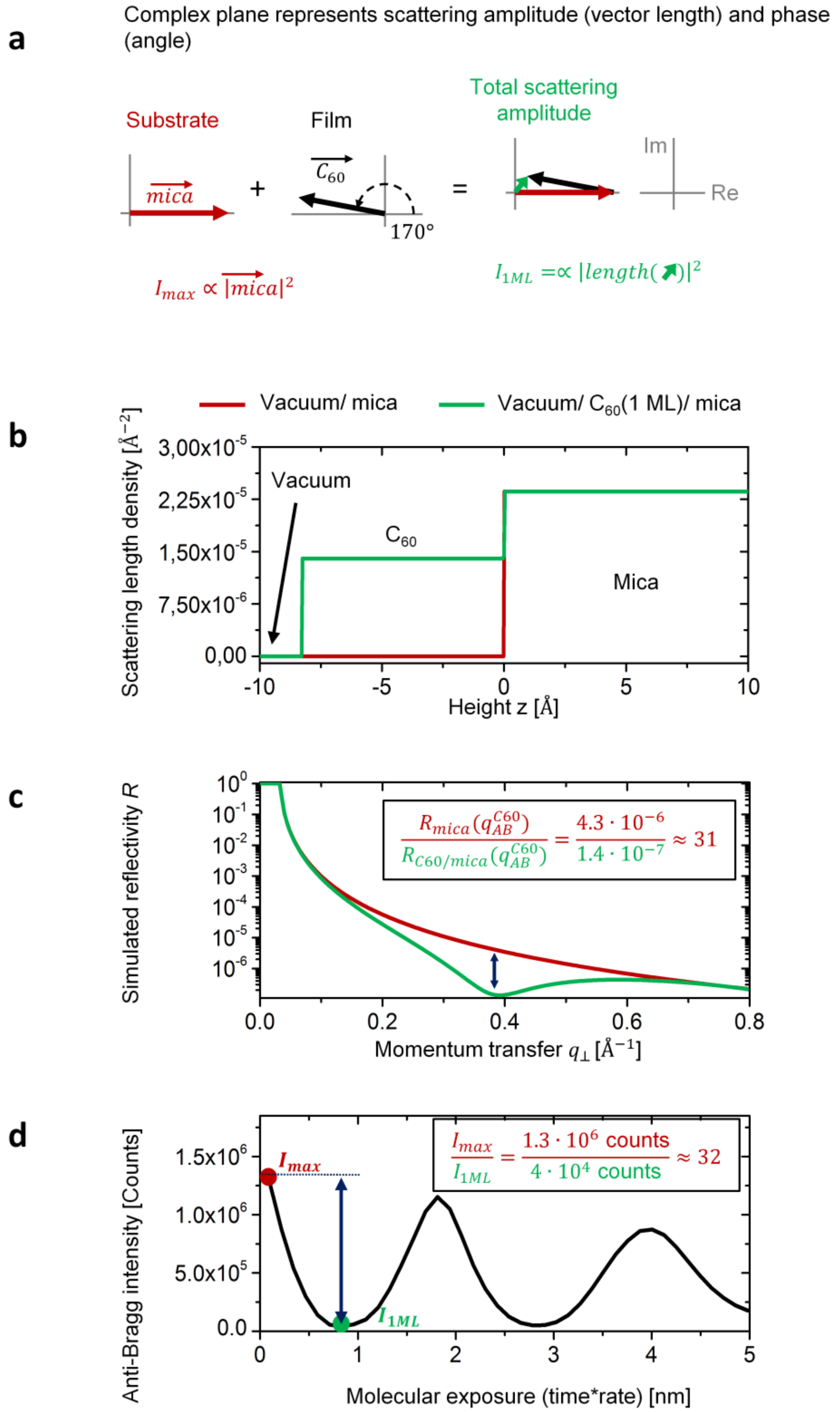

Supplementary Figure 1. | Illustration of the anti-Bragg intensity during the growth. (a) Scattering amplitude vectors in the complex plane: we find a nearly vanishing resulting anti-Bragg intensity for the first monolayer. **(b)** Scattering length density (SLD) for the calculation of the reflectivity**. (c)** Calculated reflectivity for the bare mica substrate and a $\mathbf{8.3}$ Å C_{60} film on mica. **(d)** Anti-Bragg intensity for $T = 60$ °C.

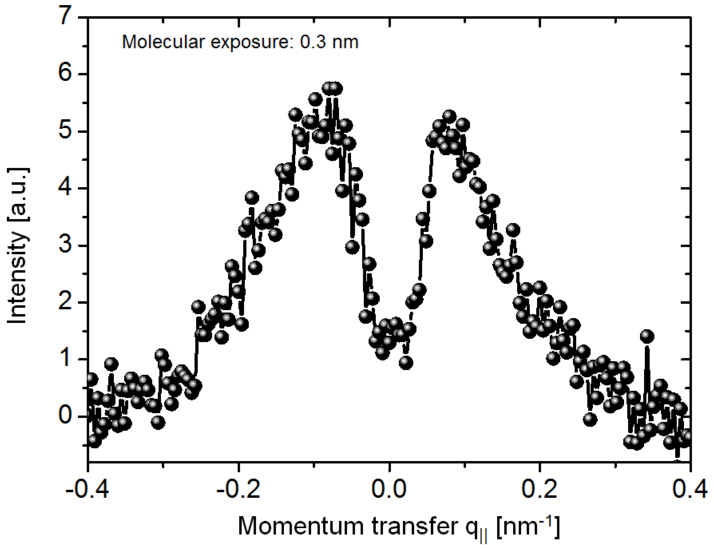

Supplementary Figure 2. | Diffusely scattered intensity for a layer coverage of 0.3 nm. This diffusely scattered intensity vs. $q_{||}$ is an extract from the (indeed very faint) colour image (main text, Fig. 2c). A clear peak splitting in the first monolayer is observed, which supports our analysis approach for the first monolayer. A clear peak splitting in the first monolayer is observed.

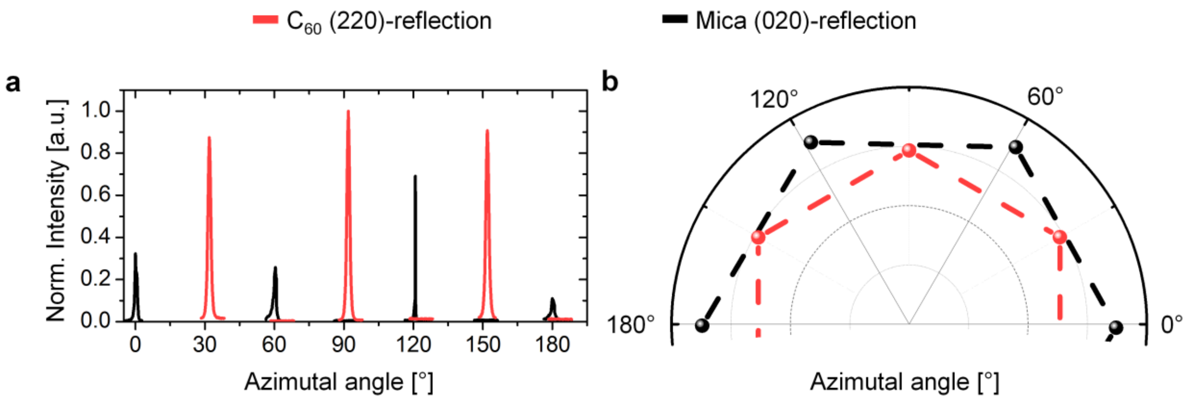

Supplementary Figure 3. | Epitaxial order of C_{60} on mica. (a) GIXD reflection of C60 and mica as a function of the azimuthal angle, **(b)** Pole figure of C_{60} and mica GIXD reflections. For the experiments a 12 nm (15 monolayer (ML)) C_{60} film on mica has been investigated. Both C_{60} and mica feature a reflection every 60°, so that we can exclude 2D powder like growth of C_{60}.

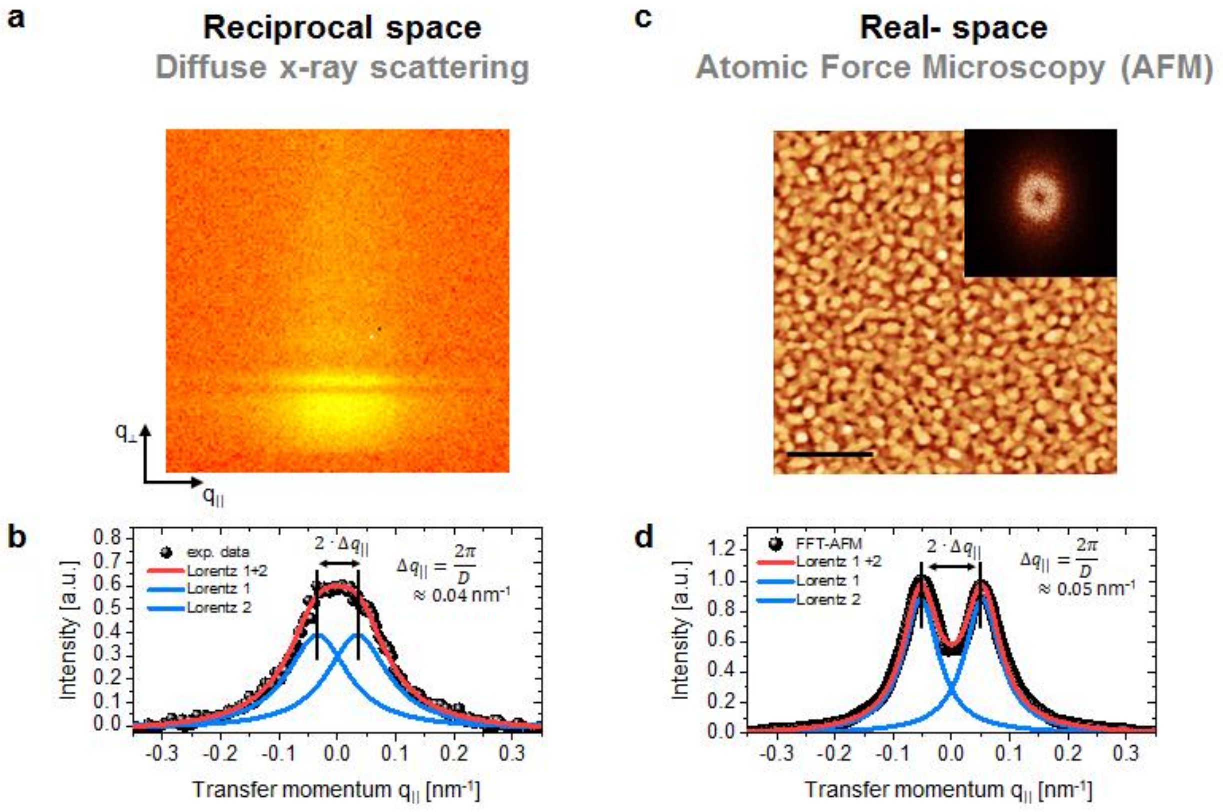

Supplementary Figure 4. | Comparison of reciprocal and real-space information. (a) Diffuse x-ray scattering (GISAXS) of a 14 ML thick C_{60} film, **(b)** line graph with Lorentz fits. **(c)** Corresponding AFM image of this film (scale bar: 500 nm) and its fast Fourier transform (FFT,inset). **(d)** Line graph of AFM Fourier transform. The good agreement between reciprocal- and real-space experiments confirms that our analysis determines correct lateral length scales.

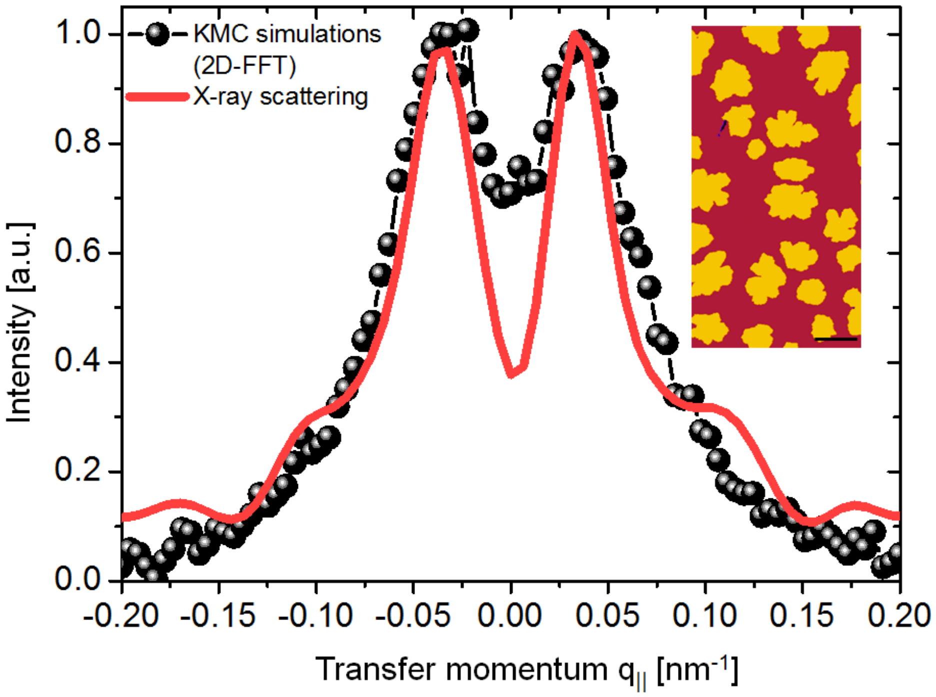

Supplementary Figure 5. | Island shapes in experiment and KMC simulations. Comparison of profile line graphs of the diffuse x-ray scattering and the fast Fourier transform (FFT) of the KMC simulated real-space morphology for a 3.5 ML C_{60}-film. Good agreement of the line graphs regarding the shape and the peak positions demonstrate agreement between island shapes in experiment and simulation. Inset: Corresponding KMC morphology (scale bar: 100 nm).

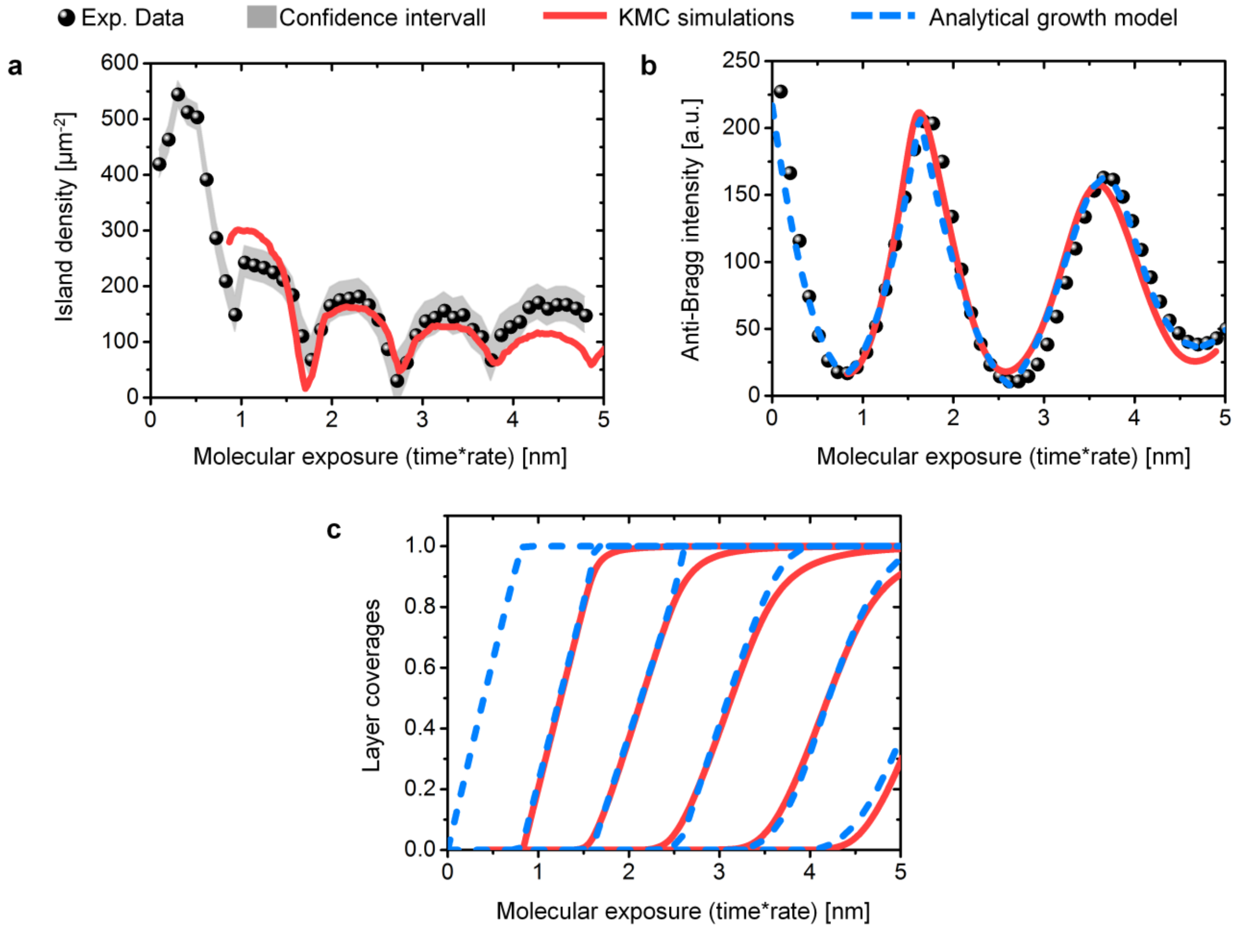

Supplementary Figure 6. | Experimental and simulated measures of the morphology for *T*=40°C. (a) Experimental and simulated island density, **(b)** anti-Bragg growth oscillations and **(c)** layer coverages for 40°C and 0.1 ML min^{-1} as a function of molecular exposure.

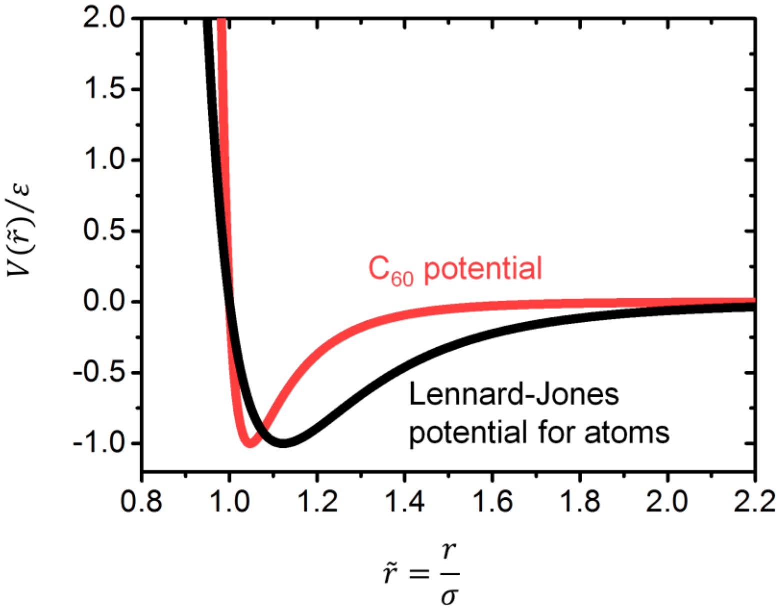

Supplementary Figure 7. | Attractive interaction range of C_{60} compared to atoms. Attractive interaction potentials for argon-atoms (black) and the fullerene C_{60} (red). Both $V(\mathrm{r})$ and r have been expressed in terms of respective values of 4ε and σ of fullerene and argon atoms, where ε is the potential minimum and σ is the distance where $V(\mathrm{r} = \sigma) = 0$.

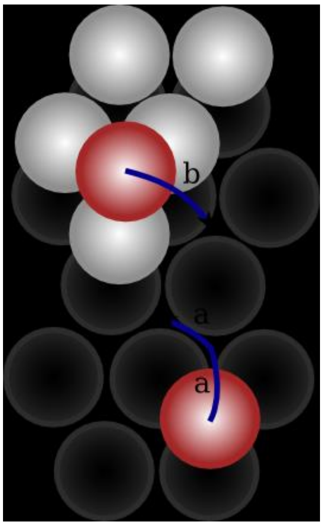

Supplementary Figure 8.| Sketch of diffusion paths. Diffusion paths during **(a)** in-plane and **(b)** step-edge crossing processes.

Supplementary Note 1: Anti-Bragg intensity during the growth of the first monolayer of C_{60} on mica

We note the curious side-observation of the C_{60} growth oscillations, where the reflectivity of the C_{60} growth oscillations reaches almost zero after deposition of one C_{60} ML (i.e. the reflected counts are reduced from $I_{\mathrm{max}} = 1.3 \cdot 10^6$ to $I_{\mathrm{1ML}} = 4 \cdot 10^4$). We emphasize that this has no particular implications for the growth itself, but we shall nevertheless briefly explain the origin of this scattering feature (see also reference [1]).

Using the formula for the Anti-Bragg oscillations given in the methods section, we fit the experimental growth oscillations and in particular the value of the minimal reflectivity at 1 ML coverage:

$$I_{\mathrm{anti-Bragg}}(1ML) = \left|A_{\mathrm{sub}}\, e^{i\varphi_{\mathrm{sub}}} + f(q_{\mathrm{z}}) \textstyle\sum_{n=1}^{1} 1 \cdot e^{i\pi n}\right|^2 \qquad (1)$$

Obviously, the scattering can vanish if the amplitudes involved are comparable and the phases are destructive (near 180°). If we insert the numbers appropriate for our system, we find indeed a nearly vanishing resulting intensity:

$$I_{\mathrm{anti-Bragg}}(1ML) \propto I_{\mathrm{max}} \cdot \left|1 \cdot e^{i\,170^\circ \frac{\pi}{180^\circ}} + 0.92\right|^2 = \frac{I_{\mathrm{max}}}{32}. \qquad (2)$$

Note that C_{60} has a scattering amplitude that is a little smaller than the mica scattering amplitude ($f(q_{\mathrm{z}}) = 0.92 A_{\mathrm{sub}}$) and has a relative phase of $\varphi = 170°$ (see Supplementary Fig. 1a for a graphical illustration of scattering amplitudes).

An alternative calculation of the absolute reflection intensities (with the same qualitative and quantitative result) uses the material densities of 1.65 g/cm^3 for C_{60}[2] and 2.83 g/cm^3 for mica[3]. From the density the mica and C_{60} scattering length density (SLD) are calculated using

$$SLD = \frac{\rho}{M} \cdot N_{\mathrm{Avogardo}} \cdot N_{\mathrm{el}} \cdot r_{\mathrm{e}} \qquad (3)$$

with the bulk material density ρ, the molar mass M (taking into account the chemical composition of mica ($KAl_3Si_3O_{12}H_2$) and the fullerene C_{60}) the number of electrons N_{el} (for $KAl_3Si_3O_{12}H_2$ and C_{60} respectively) and the Thomson scattering length $r_{\mathrm{e}} = 2.818 \cdot 10^{-5}$ Å. This gives a mica scattering length density of $2.4 \cdot 10^{-5}$ Å^{-2} in agreement with literature values[4], and for C_{60} a scattering length density of $1.4 \cdot 10^{-5}$Å^{-2} is obtained. Using these values for the SLD and a C_{60} monolayer thickness of 8.3 Å (corresponding to the C_{60} lattice constant) we arrive at SLD profiles shown in Supplementary Fig. 1b for the bare substrate (vacuum/

mica interface) and the one monolayer C_{60} on mica structure (vacuum/ C_{60} (1ML)/ mica). We use the Parratt formalism for the calculation of the reflectivity of stratified layers from the scattering density profile (see reference [5] for details of this recursive transfer matrix method).

Supplementary Fig. 1c shows the calculated x-ray reflectivity as a function of q for the bare substrate and 1ML on the substrate. For the C_{60} on mica reflectivity a pronounced dip (Kiessig fringe) can be seen at the anti-Bragg point of C_{60} ($q_{\perp} = q_{\mathrm{AB}}^{\mathrm{C60}} = 0.38\ \mathrm{Å}^{-1}$, which is equivalent to an incidence angle $\theta = 1.65°$) due to the C_{60} layer reflectivity. From the simulated reflectivity we find a reduction by a factor of 31 at the anti-Bragg point ($4.3 \cdot 10^{-6}$ for bare mica to a reflectivity of $1.4 \cdot 10^{-7}$ with one C_{60} ML) neglecting the small contribution of surface roughness in the calculation. Comparison with the experimentally observed reduction by a factor of 32 as obtained from the growth oscillations count rates for 0ML and 1ML in Supplementary Fig. 1d shows good agreement between theory and experiment.

Supplementary Note 2: Epitaxial order of C_{60} on mica

We have investigated the epitaxial order and thin film structure of C_{60} on mica using Grazing Incidence X-ray Diffraction (GIXD) performed on a rotating anode lab source (Cu-K_{α} radiation, $\lambda = 1.54$Å). This provides in-plane information on the crystallinity as a function of the lateral momentum transfer $q_{||}$. Azimuthal rotation of the sample at constant momentum transfer $q_{||}^{\mathrm{C_{60}}} = 1.254\ \mathrm{Å}^{-1}$ and $q_{||}^{\mathrm{mica}} = 1.394\ \mathrm{Å}^{-1}$ corresponding to the C_{60} (220)-reflection and mica (020)-reflection reveals that the order of C_{60} on the underlying substrate is epitaxial. In Supplementary Fig. 3a the particular GIXD reflections of C_{60} (red) and mica (black) are shown for a 12 nm thick C_{60} film as a function of the azimuthal angle. To emphasize the epitaxial order of C_{60} on top of mica(001) a pole figure can be seen in Supplementary Fig. 3b. Both C_{60} and mica feature a reflection every 60°, so that we can exclude 2D powder like growth of C_{60}. Nevertheless, multiple domains and stacking faults are possible and cannot be excluded.

Supplementary Note 3: Equivalence of real-space and reciprocal-space methods

Real-space images of the first layers using AFM are unfortunately not possible due to strong dewetting effects in the first monolayers for C_{60} on mica. Nevertheless, despite post-growth dewetting effects in the first layers, we could image the morphology of stable, thicker films with atomic force microscopy, as shown in

Supplementary Fig. 4. In the kinematic approximation, the Fourier transform (FT) of the real-space structure corresponds to the diffusely scattered intensity in our x-ray experiments[6]. Comparing the diffuse x-ray scattering, which unfortunately for the thick films is close to the resolution limit and the FT of the corresponding AFM image for an 14 ML thick film ($T = 60$ °C, high deposition rate) we find very similar peak splitting of $\Delta q_{||} \approx 0.04$ nm^{-1}, respective 0.05 nm^{-1}. The line graph of the FT was convoluted with the resolution function of the experiment (normalized Gaussian function with a FWHM of 0.01 nm^{-1}). This good agreement between reciprocal- and real-space experiments confirms that our analysis determines correct lateral length scales.

Supplementary Note 4: Island shapes in experiment and KMC simulation

In general, the island shape can be obtained from the shape of the diffuse x-ray scattering (GISAXS) as the scattered intensity is composed of the structure factor (average island distance) and the form factor (island shape) of the thin film morphology. By assuming a certain island shape and (size) distribution of islands one can calculate the diffusely scattered intensity from this film morphology using dedicated programs like IsGISAXS[7]. Here we do not have to assume island shapes, but can directly use our KMC data to calculate the expected shape of the diffusely scattered intensity. In Supplementary Fig. 5 line graphs of the diffuse x-ray scattering and the Fourier transform of the real-space morphology for a 3.5 ML thin C_{60} film (60°C, low deposition rate) are compared. The line graph of the FT was convoluted with the resolution function of the experiment (normalized Gaussian function with a FWHM of 0.01 nm^{-1}). There is good agreement between the simulation and measurement, with only slight deviations due to experimental resolution limits. This finding supports our assumption that we have similar island shapes in experiment and simulation.

Supplementary Note 5: Island density, anti-Bragg growth oscillations and layer coverages for $T = 40$ °C and $f = 0.1$ ML min^{-1}

The comparison of the experimental lateral information (island density) and vertical information (anti-Bragg growth oscillations and layer coverages) was performed for rates of 0.1 ML min^{-1} and 1 ML min^{-1} and temperatures of 40 °C, 60 °C, and 80 °C. In addition to the data for a temperature of 60°C and rate of 0.1 ML min^{-1} shown in the main text, we present in Supplementary Fig. 6a and b an additional example for a temperature of 40°C and a rate of 0.1 ML min^{-1}.

In accordance with growth theories[8] and also correctly predicted by our KMC simulations, we find experimentally that the island densities increases (that is, the island sizes decreases) with lower substrate temperature (Supplementary Fig. 6a). The anti-Bragg intensity (Supplementary Fig. 6b) for $T = 40$ °C shows distinct growth oscillations indicating layer-by-layer growth. From fitting the anti-Bragg growth oscillations using an analytical growth model[9] one can extract the layer coverage. The results support the simulated KMC layer coverages for $T = 40$ °C as can be seen in Supplementary Fig. 6c.

Supplementary Note 6: KMC parameter choice: the correlation between energy barriers

To adjust the parameters E_D, E_B and E_ES appearing in the energy barrier ($\Delta E_{i,j} = E_\mathrm{D} + n_i E_\mathrm{B} + s_{i,j} E_\mathrm{ES}$) (see equation (1) in the main text) we start from initial values suggested in the literature[10,11]. We then optimize the parameter set to match, as accurately as possible, the experimental data for the island density and the filling fraction at $T = 40$ °C and $f = 0.1$ ML min^{-1}. This experimental data set has the best lateral and temporal resolution. In performing such an optimization, we have to note that the influences of the different energy barrier contributions on our observables are strongly correlated. For example, E_D determines the free diffusion time and is therefore of prime importance for the calculated island density. The latter, however, is also influenced by E_B: If the neighbour energy is sufficiently small, island nuclei can dissociate, which effectively reduces the island density. Therefore the influence of E_D and E_B on the island is correlated. Another example occurs during step-edge crossing: The energy barrier of this process is given by E_ES. However, the effective crossing rate is also influenced by the island morphology (which determines the probability of reaching a step), and thus, by E_B. Due to this mutual influence it is clear that optimization of the energy parameters is rather challenging, one danger being that the resulting set may not be completely unique. We note, however,

that the final parameter set selected in our study (see Fig. 1 in the main text) yields a satisfying match of the experimental data not only for the case $T = 40$ °C and $f = 0.1$ ML min^{-1}, but also at the other temperatures and adsorption rates considered. This "robustness" strongly supports our predictions. For the estimation of the error bars of the extracted energies their mutual correlation as well as the experimental confidence interval was taken into account. Notably, even a small alteration of one of the energy contributions (changes of the order of 20 meV) results in significant changes of the calculated morphology, i.e., in a deviation from experimental results. The confidence interval of the experimental island density data shown in Fig. 3 in the main text is calculated from the experimental uncertainties of x-ray wavelength, sample-to-detector distance as well as the fit uncertainty of the double peak distance in the diffuse x-ray scattering experiments. When we take the experimental confidence interval of the island density and anti-Bragg intensity into account, the error bar of the diffusion barrier increases to 40 meV.

Supplementary Note 7: Discussion on the diffusion barrier E_D

Both Körner *et al.*[12] and Liu *et al.*[11] report a free diffusion energy $E_2 \approx 0.178$ eV; however,they also use an attempt frequency of $\nu_0 = 2 \cdot 10^{11}$Hz. Moreover, both studies are based on a hexagonal lattice under consideration of interstitial sites. Here, we neglect these sites, yielding a somewhat coarse-grained approach. We note that without the coarse-grained approach it would not be possible to simulate such a large system for minutes to hours of experimental time. In one diffusion step on our coarse-grained lattice a particle overcomes the barrier $E_2 \approx 0.178$ eV twice. Moreover, one has to note that there are three diffusion options from the interstitial site. Since only one option leads to our coarse-grained destination site, an additional geometric factor of 1/3 needs to be included in the diffusion rate. Furthermore, taking the difference in the attempt frequency ν_0 into account, we obtain the following estimate of a coarse-grained free diffusion barrier from the values reported in[11,12]

$$E_\mathrm{D} \approx -\ln\left(\frac{1.4\cdot 10^{13}}{2\cdot 10^{11}}\right) k_\mathrm{B}T - \ln\left(\frac{1}{3}\right) k_\mathrm{B}T + 2\cdot 0.178\ \mathrm{eV} \approx 0.51\ \mathrm{eV}. \quad (4)$$

This value lies within the error margins of our value E_D. This estimate was obtained using $T = 333$ K.

Supplementary Note 8: Attractive interaction range of C_{60} compared to atoms and colloids

The effective, centre-of-mass interaction between two C_{60} molecules decays significantly faster to zero with the (centre-of-mass) distance r than that between atoms. Specifically, the potential has the form[13,14] (as obtained based on a calculation of Girifalco[15])

$$V_{C_{60}}(\mathrm{r}) = \frac{\varepsilon_{\mathrm{C}_{60}}}{34}\left[9\left(\frac{1.047\sigma_{\mathrm{C}_{60}}}{\mathrm{r}}\right)^{43} - 43\left(\frac{1.047\sigma_{\mathrm{C}_{60}}}{\mathrm{r}}\right)^{9}\right] \qquad (5)$$

, where ε is the depth of the potential and σ the centre-of-mass separation at which the potential is zero. The distance dependence of the attractive part of the potential, $-1/r^9$, results from an angle-average over all the van-der-Waals interactions ($\sim 1/r^6$) between the individual carbon sites.

Contrary to Supplementary Equation (5), the attractive interaction between atoms can be described by a conventional Lennard-Jones potential which decreases as $-1/r^6$ for large distances:

$$V_{\mathrm{atom}}(\mathrm{r}) = 4\varepsilon_{\mathrm{atom}}\left[\left(\frac{\sigma_{\mathrm{atom}}}{\mathrm{r}}\right)^{12} - \left(\frac{\sigma_{\mathrm{atom}}}{\mathrm{r}}\right)^{6}\right]. \qquad (6)$$

To illustrate the different attraction ranges, the interaction potentials for atoms (here Argon) and C_{60} are depicted in Supplementary Fig. 7. Note the significantly shorter attractive interaction range of C_{60} compared to atoms.

An even shorter range of attractive interactions occurs in colloidal systems. Here, the attractive interaction between two colloidal particles typically originates from depletion forces induced by solvent particles. The range of the resulting attractive interaction is determined by the size of the solvent molecules, which can be orders of magnitude smaller than the size of the colloidal particles themselves[16].

Supplementary Note 9: Calculation of particle-resolved dynamics for atomic systems: comparison of the role of neighbour interactions in C_{60} and atomic systems

To isolate, within our KMC simulations, the role of neighbour interactions on the growth of representative atomic systems relative to C_{60} growth, we proceed as follows: The atom-like KMC simulations are performed with the same values of E_D, E_{ES} used in the C_{60} simulations, as well as with the same lattice configuration and experimental input parameters. However, the values for the neighbour interaction E_B of atomic systems are chosen such that the ratio $R = E_D/(E_D + E_B)$ fulfils the literature values[17] $R \approx 0.34$ for elemental atomic systems like Pt ($R \approx 0.29 - 0.34$) or Ag ($R \approx 0.29 - 0.39$). Analysing systems that are identical in all parameters except the ratio R allows us a direct comparison of single-particle dynamics despite the smaller time- and length scales of growth in atomic systems relative to C_{60}. Clearly, this strategy is not suited to make predictive simulations of the growth of the atomic systems, but it does enable us to determine the influence of the difference in range of attractive interactions.

Supplementary Note 10: Origin of Ehrlich-Schwoebel barrier: Calculation of geometric contribution

The subsequent argumentation closely follows that given by Ganapathy et al.[18] for colloidal systems. We consider a C_{60} particle moving on a surface formed by other C_{60} particles. As a consequence of the short range of interactions the travelling particle tries to be in constant contact with two other particles. This effectively reduces local transport to a one-dimensional (1D) motion along a straight path, along which the potential landscape can be assumed to be constant. We note that the length of the 1D path between binding sites (d_{island}) on an island is smaller than that of a path crossing the step-edge (d_{step}), see Supplementary Fig. 8 for an illustration of such paths. Therefore, the travel time for a step-edge jump can be up to a factor of $F = \frac{\langle t_{\text{step}} \rangle}{\langle t_{\text{island}} \rangle} = \left(\frac{d_{\text{step}}}{d_{\text{island}}}\right)^2$ longer, where $\langle t \rangle = \frac{1}{r}$ with r as the Arrhenius-type rate describing the surface processes[19]. Associated with this increase in diffusion time along the 1D potential is an increase of the probability to return to the original site[20]. As a consequence, the step-edge crossing probability effectively decreases. This consideration leads to an effective, geometrical Ehrlich-Schwoebel barrier $E_{\text{ES,geo}}$ determined by

$$E_{\mathrm{ES,geo}} = \ln\left(\left(\frac{d_{\mathrm{step}}}{d_{\mathrm{island}}}\right)^2\right) * k_{\mathrm{B}} * T. \quad (7)$$

To quantify this geometry-induced energy contribution, we recall that our KMC simulations are based on a triangular lattice. This yields for the ratio $\frac{d_{\mathrm{step}}}{d_{\mathrm{island}}}$ a value of 1.4. This value is based on the considerations made in the supplemental material of Ganapathy et al.[18], where they found for a hexagonal lattice that the path-length of local motion in a 1D potential along the path crossing a step-edge (see b in Supplementary Fig. 8) is 2.8 times as long as the path-length involving diffusion between neighbouring sites (see a in Supplementary Fig. 8). Since our simulation is restricted to a triangular lattice, an in-plane diffusion process involves two nearest-neighbour steps. This results in the ratio $\frac{d_{\mathrm{step}}}{d_{\mathrm{island}}} = \frac{b}{2a} = \frac{2.8a}{2a} = 1.4$. Combining this result with Supplementary Eq. (7) we conclude that the geometrical Ehrlich-Schwoebel barrier $E_{\mathrm{ES,eff}}$ is less than 50 meV for a temperature equal or less than 80°C.

Supplementary References

1. Kowarik, S., Gerlach, A., Skoda, M. W. A., Sellner, S. & Schreiber, F. Real-time studies of thin film growth: measurement and analysis of X-ray growth oscillations beyond the anti-Bragg point. *Eur. Phys. J. Spec. Top.* **167,** 11–18 (2009).

2. Ruoff, R., Thornton, T. & Smith, D. Density of fullerene containing soot as determined by helium pycnometry. *Chem. Phys. Lett.* **186,** 456–458 (1991).

3. Anthony, J. W., Bideaux, R. A., Bladh, K. W. & Nichols, M. C. *Handbook of Mineralogy*. (Mineralogical Society of America, 2001).

4. Jeng, U.-S. *et al.* Neutron and X-ray Scattering on the Monolayer Structure of a Lecithin Fullerene-Derivative. *J. Nanosci. Nanotechnol.* **7,** 1406–1413 (2007).

5. Als-Nielsen, J. & McMorrow, D. *Elements of Modern X-Ray Physics*. (Wiley, 2001).

6. Müller-Buschbaum, P. in *Appl. Synchrotron Light to Scatt. Diffr. Mater. Life Sci.* (Ezquerra, T. A., Garcia-Gutierrez, M. C., Nogales, A. & Gomez, M.) 61–89 (2009).

7. Schwartzkopf, M. *et al.* From atoms to layers: in situ gold cluster growth kinetics during sputter deposition. *Nanoscale* **5,** 5053–5062 (2013).

8. Michely, T. & Krug, J. *Islands, Mounds, and Atoms: Patterns and Processes in Crystal Growth Far From Equilibrium (Springer Series in Surface Sciences)*. (Springer Berlin Heidelberg, 2003).

9. Woll, A. R., Desai, T. V. & Engstrom, J. R. Quantitative modeling of in situ x-ray reflectivity during organic molecule thin film growth. *Phys. Rev. B* **84,** 075479 (2011).

10. Goose, J. E., First, E. L. & Clancy, P. Nature of step-edge barriers for small organic molecules. *Phys. Rev. B* **81,** 205310 (2010).

11. Liu, H., Lin, Z., Zhigilei, L. V. & Reinke, P. Fractal structures in fullerene layers: simulation of the growth process. *J. Phys. Chem. C* **112,** 4687–4695 (2008).

12. Körner, M. *et al.* Second-Layer Induced Island Morphologies in Thin-Film Growth of Fullerenes. *Phys. Rev. Lett.* **107,** 016101 (2011).

13. Hagen, M. H. J., Meijer, E. J., Mooij, G. C. A. M., Frenkel, D. & Lekkerkerker, H. N. W. Does C60 have a liquid phase? *Nature* **365,** 425–426 (1993).

14. Tewari, S. P., Dhingra, G. & Silotia, P. Collective Dynamics of a Nano-Fluid: Fullerene, C60. *Int. J. Mod. Phys. B* **24,** 4281–4292 (2010).

15. Girifalco, L. Interaction potential for C60 molecules. *J. Phys. Chem.* **2,** 5370–5371 (1991).

16. Likos, C. Effective interactions in soft condensed matter physics. *Phys. Rep.* **348,** 267–439 (2001).

17. Evans, J. W., Thiel, P. A. & Bartelt, M. C. Morphological evolution during epitaxial thin film growth: Formation of 2D islands and 3D mounds. *Surf. Sci. Rep.* **61,** 1–128 (2006).

18. Ganapathy, R., Buckley, M. R., Gerbode, S. J. & Cohen, I. Direct measurements of island growth and step-edge barriers in colloidal epitaxy. *Science* **327,** 445–448 (2010).

19. Reimann, P., Schmid, G. & Hänggi, P. Universal equivalence of mean first-passage time and Kramers rate. *Phys. Rev. E* **60,** R1–R4 (1999).

20. Pólya, G. Über eine Aufgabe der Wahrscheinlichkeitsrechnung betreffend die Irrfahrt im Straßennetz. *Math. Ann.* **84,** 149 – 160 (1921).
